# *Chlamydia pneumoniae *aggravates vein graft intimal hyperplasia in a rat model

**DOI:** 10.1186/1471-2180-7-111

**Published:** 2007-12-06

**Authors:** Geoffrey TL Kloppenburg, Rick de Graaf, Gert ELM Grauls, Cathrien A Bruggeman, Frank R Stassen

**Affiliations:** 1 Cardiovascular Research Institute Maastricht, Department of Medical Microbiology, Academic Hospital Maastricht/Maastricht University, Maastricht, MD Maastricht, The Netherlands; 2 Cardiovascular Research Institute Maastricht, Department of Radiology, Academic Hospital Maastricht/Maastricht University, Maastricht, P.O. Box 616 6200 MD Maastricht, The Netherlands

## Abstract

**Background:**

Along with angioplasty, autologus vein grafts are commonly used for artery bypass grafting in patients with advanced arterial stenosis and drug-resistant angina pectoris. Although initially a successful procedure, long-term functionality is limited due to proliferation and migration of smooth muscle cells. Like in atherosclerosis, common chronic infections caused by viruses and bacteria may contribute to this process of vein graft failure. Here we investigated the possible role of *Chlamydia pneumoniae *(*Cpn*) in the pathogenesis of venous graft failure in an experimental animal model. In 2 groups (n = 10 rats/group), an epigastric vein-to-common femoral artery interposition graft was placed. Immediately thereafter, rats were infected with *Cpn *(5*10^8 ^IFU) or injected with control solutions. Rats were sacrificed three weeks after surgery and the grafts were harvested for morphometrical and immunohistochemical analysis.

**Results:**

*Cpn *administration immediately after vein grafting resulted in a significant increase in medial cross-sectional area, wall thickness and total wall area. There were no significant differences in T-cell or macrophage influx. Likewise, although positive immunostaining for both HSP60 and CRP could be detected, no differences were found between groups. Based on the observation that the number of cells/μm^2 ^was also not altered, we conclude that Cpn infection stimulates smooth muscle cell proliferation by hereunto unknown molecular mechanisms, resulting in a significant increase in intimal hyperplasia.

**Conclusion:**

In conclusion, in a well defined animal model we present here for the first time evidence for a role of *Chlamydia pneumoniae *in the process of venous graft failure.

## Background

Besides internal mammary arteries, autologus saphenous vein grafts are commonly used for coronary artery bypass grafting (CABG) in angina pectoris patients, resistant to aggressive medical therapy or patients with advanced coronary artery stenosis not suitable for percutaneous transluminal coronary angioplasty (PTCA). Although the initial results of venous grafts are excellent, the symptoms tend to recur due to vein graft degeneration and stenosis greatly limiting the long term success of bypass surgery. Early failure occurs within the first 1 to 2 months probably from primary thrombosis often due to technical failure or poor runoff in severely stenotic distal coronary arteries [1]. Late failure occurs from several months to years after bypass surgery and is caused by neointimal hyperplasia (NIH) with subsequent atherosclerosis in the saphenous vein graft [2]. NIH, defined as the accumulation of phenotypically altered medial smooth muscle cells (SMC) and extracellular matrix in the intimal component of the vein, is most prominent in the venous graft within the first year. Several factors interact to influence the development of NIH mostly initiated by ischemia of the venous wall, mechanical trauma and hemodynamic stress. Moreover, convincing evidence suggested that NIH is associated with extensive endothelial denudation and destruction of venous grafts [3], resembling a response to injury as seen after angioplasty often leading to restenosis of the denuded artery.

Today it is generally accepted that atherosclerosis is an inflammatory diseases and that atherogenesis as well as disease progression result from inflammation and immune responses towards various stimuli. Common chronic infections caused by viruses and bacteria have been suggested to contribute to this inflammatory process. Most compelling evidence comes from data concerning the intracellular pathogen *Chlamydia pneumonia *(*Cpn*). Although this bacterium was initially identified as a causative factor in (a-)symptomatic inflammation of the airways, clinical studies demonstrated that patients with high titers of antibody against *Cpn *have an increased risk for cardiovascular complications [4,5]. This is supported by studies revealing significant accelerations of lesion development in animals and a large variety of pro-atherogenic effects in vitro [6-8]. In addition, recent studies have shown that *Cpn *infection promotes a proliferative phenotype in the vasculature, which makes *Cpn *also a likely risk factor for vein graft failure [9,10]. This is supported by the work of Bartels et al., who observed a strong correlation between elevated *Cpn *IgG titers and the detection of *Cpn *in occluded vein grafts [11]. Similar data were shown by Zorc et al., implicating a possible relationship between *Cpn *presence and occluded arterial bypass grafts [12]. These studies suggest that *Cpn *is present in occluded grafts, however, experimental evidence for this is currently lacking. In the present paper we addressed this by examining the contribution of *Cpn *to neointimal hyperplasia in a well defined animal model for autologous vein grafting. To our knowledge this is the first paper which addresses the possible contribution of *Cpn *infection to the pathogenesis of venous graft failure in an experimental model.

## Results

### Animal condition and grafts

Starting body weight ranged from 250 g to 350 g, while at the end of the experimental period animal weight ranged from 320 g to 400 g. No differences in body weight were observed between experimental groups. During the experimental phase no apparent clinical signs of illness were observed in any of the animals. The overall graft patency after three weeks was 90%. Early thrombosis seemed the underlying cause of the failed grafts. No structural anomalies at the anastomotic regions of the grafts were observed.

### Chlamydia pneumoniae aggravates intimal hyperplasia through stimulation of SMC proliferation

**Figure 1:**
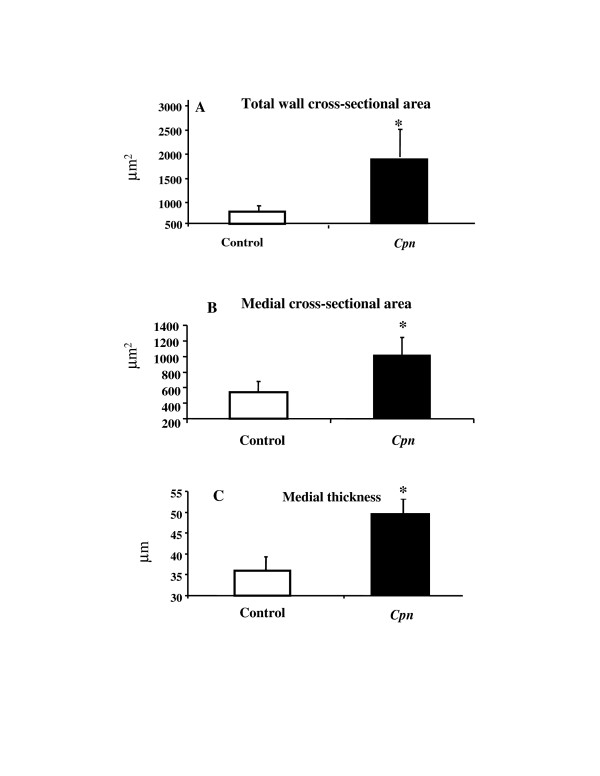
Effect of Cpn administration on total wall (A) cross-sectional area, medial cross-sectional area (B) and wall thickness (C) three weeks after vein grafting. * P < 0.05 (values compared with control).

Three weeks after bypass grafting in all veins a significant increase in medial thickness was observed (medial thickness in normal, non-grafted vein: 10 ± 2 μm vs. 36 ± 3 μm in grafted vein of the control group) and showed neointimal thickening, which is principally absent in non-grafted control veins.*Cpn *administration immediately after vein grafting resulted in a significant increase in the medial thickness (*Cpn*-group: 50 ± 3 μm vs. control group: 36 ± 3 μm, p ≤ 0.05), total wall cross-sectional area (*Cpn*-group: 184200 ± 57932 μm^2 ^vs. control group: 75359 ± 15473 μm^2^, p ≤ 0.05) and medial cross-sectional area (*Cpn*-group: 101731 ± 22984 μm^2 ^vs. control group: 54149 ± 13960 μm^2^, p ≤ 0.05) at 3 weeks post surgery (Figure 1).

Next, we tried to determine whether the increase in cross-sectional area results from a concomitant increase in cell number or whether the increase is due to cellular hypertrophy, interstitial oedema or an increase in extracellular matrix components. Therefore, we determined cellular density. Our finding that the average number of cells/μm^2 ^was not significantly different between groups (*Cpn*-group: 5.0 ± 0.7 × 10^-3 ^cells/μm^2 ^vs. control group: 4.1 ± 0.5 × 10^-3 ^cells/μm^2^), suggests that *Cpn *infection stimulates cell proliferation but not cellular hypertrophy or interstitial oedema or an increase in extracellular matrix components. After all, the latter are expected to reduce cellular density, a phenomenon which was not observed.

### Influx of inflammatory cells

Staining for inflammatory cells in the venous graft showed a minimal influx of T-cells at three weeks after surgery with an average of one to three T-cells per cross-section. No significant differences between experimental groups were observed. On the other hand, macrophages were notably present in the subendothelial area and in the media, however with a with a wide variance. Therefore, no significant differences between groups were found. Moreover, alpha-actin staining showed SMC to be the main content of the intima and media with little extra-cellular matrix accumulation in both groups, confirming that the increase in wall mass is predominantly due to smooth muscle cell proliferation.

### C-reactive protein

**Figure 2:**
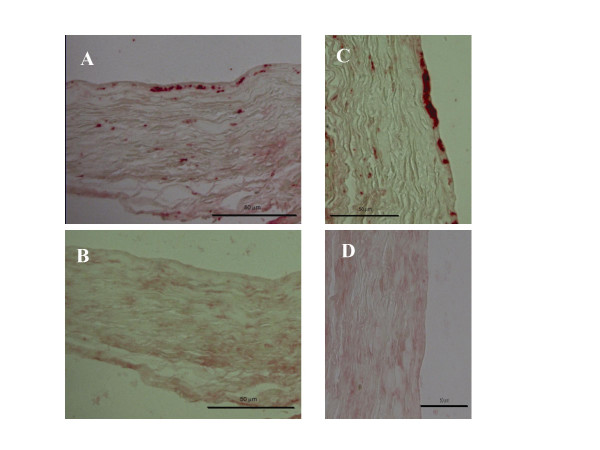
Photomicrographs showing subsequent immunolabeling of a venous bypass graft 3 weeks after surgery (A: CRP, C:HSP60) as well as their representative negative controls (B, D).

We also performed an immunostaining to reveal the presence of C-reactive protein (CRP) in the venous graft. Although predominantly produced by the liver, local production of CRP by SMC in response to inflammatory cytokines has also been demonstrated recently [13]. Also, evidence suggests that CRP is involved in smooth muscle cell migration and proliferation [14]. Truly, positive staining for CRP could be detected mainly in the subendothelial area (typical example shown in Figure 2A). Nonetheless, when stainings were quantified in a semi-quantitative manner, no significant differences were observed between groups (*Cpn*-infected: 2.5 ± 0.8 v.s. control group 2.4 ± 0.5, n.s.).

### Heat shock protein 60

Previously we demonstrated the noticeable presence of heat shock protein 60 (HSP60) in occluded veins [15]. Furthermore, we were able to demonstrate that HSP60 is able to stimulate SMC proliferation in a TLR2/4 dependent way [15]. To determine whether HSP60 also plays a role in graft failure in the present model and whether *Cpn *affects the presence of HSP60 in the grafts, we stained HSP60 in grafts from both the control group and the *Cpn*-infected group. Indeed, HSP60 positive staining could be revealed in both groups (typical example shown in Figure 2C). Also, semi-quantitative analysis showed a trend towards a more intense HSP60 signal in the *Cpn*-infected group (*Cpn*-group: 3.3 ± 0.5 v.s. control group: 2.8 ± 0.7, n.s.), although the difference was not statistically significant.

## Discussion

Saphenous vein graft failure is a common problem after CABG. Approximately 15% to 30% of vein grafts occlude during the first year increasing up to 50% within 10 years [16]. Graft failure occurring within one month after surgery is almost always caused by thrombosis. Neointimal hyperplasia as defined as the accumulation of SMC and extracellular matrix in the intimal layer follows a similar pattern in the balloon injured artery or vein graft and is the major disease process in the vein graft resulting in failure during the first year [17]. Nearly all veins implanted into the arterial circulation develop intimal wall thickening thereby reducing the lumen size. This is confirmed by our results in the rat venous graft model. At 3 weeks post transplantation, a significant increase in both total and medial cross-sectional area as well as media thickness was observed in the epigastric vein grafts, which is in agreement with earlier data from Hoch and colleagues [18].

To unravel the underlying molecular mechanisms contributing to the pathology of vein graft failure a large variety of studies have been carried out of the past decades in order to find an effective intervention. However, until now these mechanisms haven't been completely elucidated yet. Recently, sero-epidemiologic studies have suggested a role for *Cpn *the development of vascular disease. *Cpn *is an obligatory intracellular Gram-negative bacterium that was first isolated as a respiratory pathogen. This micro-organism has also been isolated from coronary arteries of patients with acute coronary syndrome [12], as well as from carotid arteries [19], the aorta and peripheral arteries [20]. These observations in combination with multiple *in vitro *findings [7] demonstrating a stimulating effect of *Cpn *on SMC proliferation, supported a role for *Cpn *in obstructive vascular disease. Nonetheless, in contrast to atherosclerosis, a possible role of *Cpn *in venous bypass failure has been less subject of study. Bartels *et al.*[21] studied the prevalence of *Cpn *in occluded vein grafts and native saphenous veins and showed that *Cpn *was frequently present in occluded CABG but not in normal veins. *Cpn *DNA could be detected by PCR in 25% of the cases, while viable *Cpn *was recovered from 16% of occluded vein grafts. In this study we present for the first time evidence for a role for *Cpn *in venous graft failure.

When rats were infected with *Cpn *immediately after surgery, a significant increase in media thickness and total wall cross-sectional area was observed in the epigastric vein-to-common femoral artery graft. To unravel a potential mechanism which might explain the effect of Cpn on intima hyperplasia, we first evaluated the presence of inflammatory cells in the graft. Focal endothelial disruption, occurring during all vein graft harvesting, leads to adherence of platelets and monocytes. Several lines of evidence suggest that *Cpn *replicate within alveolar cells and can make its way into the wall of the healing graft by using macrophages and monocytes as vectors [22]. Once present in the vascular wall, *Cpn *may further enhance the influx of monocytes through the release of monocyte chemoattractant protein 1 by VSMC's. Chronic macrophage infection contributes to local inflammation but it remains unclear whether this aggravates intimal hyperplasia and bypass failure. Hoch et al. demonstrated that depletion of macrophages suppresses intimal hyperplasia indicating the importance of the amount of macrophages present in the graft [18]. However immunostaining of the venous graft in our model showed no differences in influx of inflammatory cells between the *Cpn *infected and the control group, suggesting a different mechanism of action.

*Cpn *infection of vascular smooth muscle cells increases interleukin-6 (IL-6) secretion in vitro [23]. In vivo, IL-6 is a strong inducer of liver C-reactive protein (CRP) production which is able to stimulate endothelial and smooth muscle cell proliferation providing a pathway for *Cpn *to aggravate vein graft failure [24]. In addition, evidence is accumulating suggesting that SMC are able to produce CRP and stimulate SMC migration and proliferation in an autocrine fashion [14]. Indeed, we were able to demonstrate the presence of CRP in particular in the sub-endothelial layer thereby providing further evidence for a role for CRP in intima hyperplasia. Nonetheless, no difference between the two groups were observed indicating that *Cpn *infection does not have an effect on the presence of CRP in the vascular wall at the time point examined. Nevertheless, this does not exclude that *Cpn *may affect the production of CRP at earlier time points post surgery and contribute as such to the process already at an earlier stage. Further studies are required to unravel this aspect in more detail.

*Cpn *is also known to produces large amounts of heat shock protein 60 (HSP 60) during chronic, persistent infections [25]. *Cpn *HSP60, which is well conserved during evolution and shows a high degree of homology with mammalian HSP60, is able to activate vascular endothelium, smooth muscle cells, and macrophages [26]. HSP60 is also able to stimulate SMC proliferation [15,27] and may hereby aggravate a hyperplastic response as seen in our model. Therefore, we decided to determine the presence of HSP60 by immunohistochemistry. Actually we were well able to visualize the presence of HSP60 in the grafts of both groups; however, despite the fact that there was a trend towards an increase in the presence of HSP 60 in the venous grafts of the *Cpn*-infected group as compared to the controls, this failed to reach statistical significance.

## Conclusion

Summarizing, we present here for the first time evidence for a possible role of *Chlamydia pneumoniae *in the process of venous graft failure. In a well defined animal model, a significant increase in both total and media cross-sectional area as well as media thickness was observed in vein grafts obtained from *Cpn*-infected rats, suggesting that *Cpn *is able to promote the process of intima hyperplasia. Furthermore, cellular density in the vascular wall remained constant indicating that *Cpn *stimulates cell proliferation but not hypertrophy nor the formation of institial oedema or the deposition of extracellular matrix components. In addition, we analyzed in more details some of the possible molecular mechanisms which may contribute to the observed *Cpn*-mediated effects. However, none of the suggested mechanisms (enhanced influx of inflammatory cells, augmented production of CRP or HSP60) seemed primarily responsible for the observed effects and further research is warranted to elucidate in more detail how *Cpn *affects vein graft failure.

## Methods

### Animals and Vein Grafting Procedure

Male inbred specific pathogen free (SPF) male Lewis (LEW) rats were obtained form the Department of Experimental Animal service of the University of Maastricht, the Netherlands. Experiments were carried out on animals aged 12 weeks weighing 250–350 gram. Housing and care of the animals, and all the procedures used in this study were approved by the Ethical Committee for the Use of Experimental Animals of the institution, and conform the Guide for the Care and the Use of Laboratory Animals, published by the US National Institute of Health (NIH Publication No. 85-23, revised 1985). Rats were fed standard rat chow and tap water at libitum. All surgical procedures were performed under general anesthesia and using sterile techniques. Epigastric vein-to-common femoral artery interposition grafts were placed in rats in a similar way as previously described by Hoch *et al.*[28]. In brief, each animal was anesthetized with an intraperitoneal (i.p.) injection of pentobarbital sodium (60 mg/kg). An 8 mm segment of ipsilateral epigastric vein was carefully harvested, gently irrigated with heparinized saline solution (100 U/ml), and placed as reverse interposition graft into a segmental 3 mm defect of the common femoral artery with 8 to 10 interrupted sutures of 11-0 nylon (Ethicon). The entire procedure was carried out with standard microsurgical techniques. The total ischemic time was kept to less than 30 min. Graft patency, defined as a condition of flow through the graft, was verified by visual inspection at the end of the surgical procedure.

### Experimental Design

Two experimental groups were used. Directly after the grafting procedure, rats (n = 10 animals/group) were either *Cpn-*infected by i.p. injection (1 ml of 5 × 10^8 ^inclusion forming units (IFU) dissolved in a sucrose-phosphate-glucose solution) or received a 1 ml i.p. injection of a sucrose-phosphate-glucose solution. The i.p. inoculation route instead of the intranasal route was used because of the high infectious dosage required in rat experiments. Previous experiments done in our laboratory have demonstrated no significant differences in systemic dissemination of *Cpn *between intranasal and i.p. injection (Ezzahiri, personal communication).

### Histological and morphometrical procedures

Three weeks after surgery, rats were anaesthetized, the chest and abdominal cavities were opened and a catheter was inserted into the apex of the heart. Vessels were initially flushed with physiological salt solution and then perfusion fixed with 3,7% formaldehyde in phosphate-buffered saline (PBS, pH 7.4) at physiological pressure (100 mmHg). Vein grafts were removed and fixed overnight in the same fixative and routinely processed for paraffin embedding. Cross-sections (4 μm) were haematoxylin-eosin or Lawson stained for morphometrical analysis (average of three cross-sections per graft). Intimal and medial areas were quantified using a computer-assisted morphometry system (analySIS^®^, Soft Imaging System, GmbH). The cross-sectional area of the media was defined as the area surrounded by the external and internal elastic lamina. The neointimal cross-sectional area was defined by the area surrounded by the internal elastic lamina and the arterial lumen. Final scores were given as means ± SEM.

### Quantification of cell number in the intima and media

The number of cells in the intima and media was quantified by counting the total number of nuclei using a microscope. All nuclei were counted in haematoxylin-eosin stained cross-sections. Final scores were expressed as number of cells per area and values are given as means ± S.E.M

### Immunohistochemistry

Paraffin sections (4 μm), taken from vein grafts three weeks after surgery, were routinely processed and stained with the two-layer indirect immunoperoxidase technique using monoclonal antibodies (mAb).

The following mAbs were used in this study: Anti-rat CD3 (Sera-lab, Crawley Down, UK), a mouse mAb against T-cells; anti-α smooth muscle actin (ASMA, Sigma, Missouri, USA), ED-1, a mouse mAb to monocytes/macrophages (kindly supplied by Dr. A.M. Duijvestijn Dept. of Immunology, University Maastricht, the Netherlands), a rabbit anti-HSP60 (CST, Danvers, USA) and anti-CRP (R&D systems, Minneapolis, USA) a mouse mAb against C-reactive protein.

Sections were incubated with 2% BSA/PBS (ED-1, CRP and ASMA) for 15 min at room temperature and treated with antigen retrieval buffer (CD3) for 25 min at 95°C [13]. Sections for HSP60 staining were incubated with 10 mM sodium citrate buffer pH 6.0 and microwave boiled for 10 min at 95–99°C. Monoclonals were diluted (ED-1 1/20, ASMA 1/1500, CD3 1/400, CRP 1/100) in PBS and applied to the slides for 60 min at 37°C. HSP60 monoclonals were diluted 1/100 in PBS and applied to the slides overnight at 4°C. After three wash steps with PBS for 5 min, a biotinylated goat anti-mouse secondary antibody (1/1200, DAKO Glostrup Denmark) was applied for 30 min at room temperature. Finally, sections were incubated with alkaline phosphatase-coupled streptavidin (ABC reagent, Vector Laboratories), followed by immunodetection using fast red as a substrate. To visualize nuclei, sections were counterstained with haematoxylin. Then, the presence of positive cells was analyzed by microscopy and scored semi-quantitatively, using a zero to four scale with zero meaning no positive signal and 4 extremely positive, by an observer blinded to the experimental groups. Final values were expressed as mean ± S.E.M.

### Statistical analysis

Morphometrical data and cell count numbers are expressed as means ± S.E.M. Values were compared using the Mann Whitney U test and p < 0.05 was considered as statistically significant.

## Authors' contributions

GK: surgical procedures, morphometric analyses, drafting the manuscript

RdG: immunohistochemistry

GG: semi-quantitative analyses of immunohistochemical stainings, culturing of *Chlamydia pneumoniae*

CB: participated in the study design and coordination

FS: participated in the study design, coordination and in drafting the manuscript

All authors read and approved the final manuscript.
